# Two-Year Results of an Open-Label Randomized Comparison of Everolimus-Eluting Stents and Sirolimus-Eluting Stents

**DOI:** 10.1371/journal.pone.0064424

**Published:** 2013-06-03

**Authors:** Matthijs A. Velders, Sjoerd H. Hofma, Jan Brouwer, Cees Jan de Vries, Michel Queré, Adrianus J. van Boven

**Affiliations:** 1 Medical Center Leeuwarden, Leeuwarden, The Netherlands; 2 Leiden University Medical Center, Leiden, The Netherlands; Sapienza University of Rome, Italy

## Abstract

**Background:**

Second generation drug-eluting stents were developed to improve the safety and efficacy of first generation stents. So far, limited long term randomized data exist comparing the second generation everolimus-eluting stents (EES) with first generation sirolimus-eluting stents (SES).

**Methods:**

A prospective, open-label, randomized, single center trial comparing EES and SES in all-comer patients. The primary endpoint was a composite of cardiac mortality, myocardial infarction and target vessel revascularization. Secondary endpoints included individual components of the composite, along with target lesion revascularization and stent thrombosis.

**Results:**

In total, 977 patients were randomized, of which 498 patients to EES and 479 to SES. Average age was 65.2±11.2 years and 71.6% of the population was male. Fifty percent of patients were treated for acute coronary syndrome, more often for ST-elevation myocardial infarctions in EES patients (13.7% vs. 9.2% in SES). In contrast, SES patients more often had prior interventions and showed more calcified lesions. Two-year follow-up was available in 98% of patients. The primary endpoint occurred in 10.7% of EES patients compared to 10.6% of SES patients (HR 1.00, 95% CI 0.68–1.48). Additionally, secondary endpoints were similar between groups. The rate of stent thrombosis was low for both stent types.

**Conclusion:**

In this all-comer population, there were no differences in endpoints between EES and SES during two-year follow-up. Stent thrombosis rates were low, supporting the safety of drug-eluting stent appliance in clinical practice.

**Trial registration:**

TrialRegister.nl NTR3170

## Introduction

First generation drug-eluting stents (DES) have reduced the need for revascularization procedures compared to bare-metal stents [1]. However, introduction of DES did not lead to reductions in mortality and re-infarctions but instead was associated with a higher incidence of late stent thrombosis (ST) [2], [3]. In order to improve the safety and efficacy of DES, second generation stents were developed. Everolimus-eluting stents (EES) have shown superior outcomes compared to first generation paclitaxel-eluting stents in a wide range of coronary lesions [4], [5]. Data is starting to accumulate for the comparison of EES with first generation and previous golden standard sirolimus-eluting stent (SES) but randomized data with long term follow-up are limited [6], [7].

Our goal was to compare the safety and efficacy of the second generation EES with first generation SES in all-comer patients undergoing percutaneous coronary intervention (PCI) during two year follow-up.

## Methods

The APPENDIX-AMI trial was a single center, prospective, open-label, randomized clinical superiority trial (*NTR3170,* http://www.trialregister.nl/trialreg/admin/rctview.asp? *TC = 3170*) comparing EES (Xience V [Abbott Vascular, Santa Clara, California]) and SES (Cypher [Cordis, Bridgewater, New Jersey]) in patients treated with PCI for any indication. APPENDIX-AMI was a sub-study of the XAMI trial [8], and was designed to evaluate the superiority of EES over SES in all-comer patients up to two year follow-up. The protocol for this trial and supporting CONSORT checklist are available as supporting information; see Checklist S1 and Protocol S1.

Inclusion ran between 18 September 2007 and 27 May 2010 in the Medical Center Leeuwarden, the Netherlands. Patients had to be eligible for coronary revascularization by PCI and willing to sign informed consent to be entered in the trial. Exclusion criteria were: minor patients, intravenous drug or alcohol abusers, patients unable to give informed consent, patients with a known allergy for everolimus or sirolimus, patients with known intolerance or contra-indications for acetylsalicylic acid or clopidogrel and finally patients in whom stent implantation was not deemed technically possible. Also, patients included in XAMI were not eligible for inclusion in APPENDIX-AMI. Patients were randomized in a 1∶1 fashion to EES or SES directly after angiography using sealed envelopes by research nurses. The randomization sequence was based on date of birth, resulting in different stent allocation between uneven and even dates. Operators were not blinded to the allocated stent.

### Sample Size

In the power analysis performed for the XAMI trial, a primary endpoint rate of 8% in both stent groups at 1-year follow-up was assumed. An absolute difference in the primary endpoint between the two stents of 6% at 1 year was accepted, which required in total 600 patients when assuming a power of 80%, an alpha of 0.05 and 2∶1 randomization to EES versus SES (Farr. & Mann testing method). The population size of 2000 in APPENDIX-AMI was deduced from the power analysis of the XAMI study and was estimated to be able to show a relevant difference in the rate of the primary endpoint between the groups.

Inclusion was stopped before reaching 2000 patients after exceeding the planned inclusion period due to a slower than anticipated inclusion.

### Ethics Statement

The study protocol was approved by the local ethics committee of the Medical Center Leeuwarden and the trial was conducted according to the principles of the Declaration of Helsinki. All patients gave oral consent before enrollment and written informed consent after procedure. Note: APPENDIX-AMI was primarily registered as a sub-study under XAMI (*NTR1123,* http://www.trialregister.nl/trialreg/admin/rctview.asp?TC=1123 *).* Because enrollment for APPENDIX-AMI started earlier than for XAMI, the start of the inclusion period pre-dated the registration of the XAMI study by 1 month.

### Procedure

Patients were pretreated with loading doses of aspirin and clopidogrel, in addition to intravenous heparin bolus of 5.000 IE in case of acute myocardial infarction. Interventions were performed according to local practice by high-volume operators. The use of glycoprotein IIb/IIIa inhibitors, thrombus aspiration and balloon pre-dilatation were left up to the discretion of the operator. Aspirin was recommended for life and clopidogrel for a minimum of 1 year.

### Follow-up

Protocol-defined follow-up was performed after thirty days, one year and two years by either questionnaires or phone contact. Follow-up was gathered by research nurses in a blinded fashion. Final event adjudication was performed between physicians on a consensus-basis in an unblinded fashion. No routine angiographic follow-up was scheduled. Information about in-hospital outcome was obtained from the institutional clinical database and by review of hospital records of those discharged to referring hospitals. Patient data were collected on case report forms and entered into an online database. Follow-up was planned for three years.

### Study Endpoints and Definitions

The primary endpoint was a composite of cardiac death, myocardial infarction (MI) and target vessel revascularization (TVR). MI was defined as a rise of creatine kinase (CK) more than 3 times the upper limit of normal along with a rise in CK-MB with recurrent symptoms and/or new electrocardiographic changes. In acute coronary syndrome patients, re-infarction within 48 hours after index procedure was defined as a re-elevation of CK of >1.5 times the previous value with elevation of CK-MB, along with recurrent symptoms and/or new electrocardiographic changes. MI around CABG required a CK rise of >5 times the upper limit or normal. TVR was defined as any repeat percutaneous or surgical intervention on any segment of the target vessel. Secondary endpoints included the individual components of the primary endpoint (cardiac mortality, MI or TVR), in addition to all-cause mortality, target lesion revascularization (TLR) and definite or probable ST. TLR was defined as any repeat intervention or bypass grafting of the target lesion previously treated with stenting along with the 5 mm proximal or distal vessel. ST was defined in accordance with the Academic Research Consortium definitions [9].

### Statistical Analyses

Study outcomes were analyzed using both intention-to-treat and per-protocol principle. Continuous variables are presented as means with standard deviations or medians with interquartile range (IQR) and were compared using Student’s t-test. Categorical variables are expressed as counts and percentages and were compared by means of Pearson’s χ^2^ test. All statistical tests were 2-tailed and a p-value <0.05 was considered statistically significant. Time-to-event analyses were performed using Kaplan Meier curves and survival curves were compared using log-rank tests. Finally, cox proportional hazards analyses were performed to calculate unadjusted and adjusted effect sizes. Adjustment for misbalance between the arms was done through multivariable models which included clinical characteristics that significantly differed at baseline. In case of limited events, the clinical characteristics with the strongest effect size were entered into the multivariable models to avoid over-adjustment.

## Results

**Figure 1:**
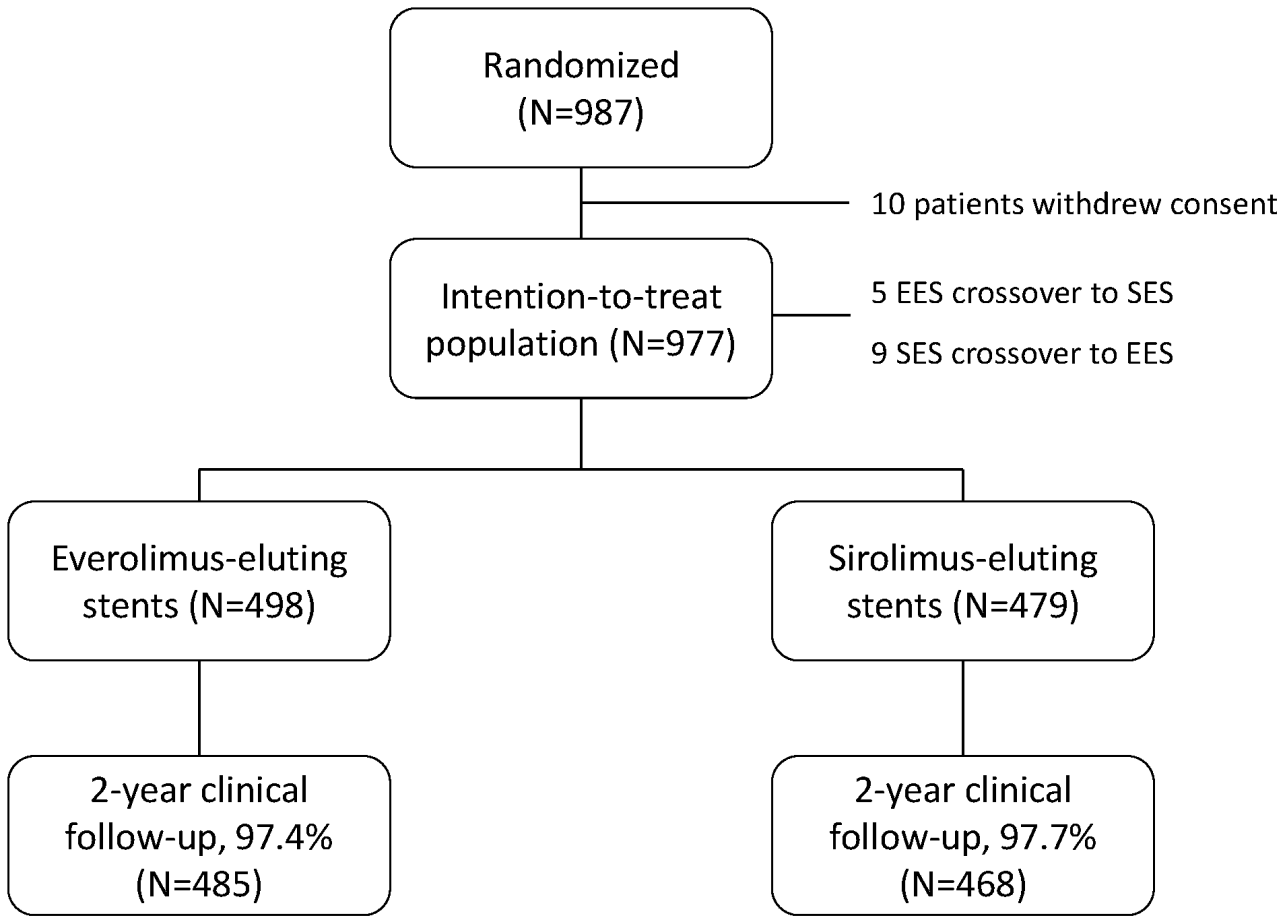
Study flowchart.

In total, 977 patients were included in the intention-to-treat analysis; 498 patients were randomized to EES and 479 patients to SES. The study flowchart is shown in Figure 1. Baseline characteristics (Table 1) showed that the average age was 65.2±11.2 years and 71.6% of the population was male. Patients in the EES arm less commonly suffered a history of hypertension and also less frequently had undergone previous PCI or coronary artery bypass grafting compared to SES patients. Furthermore, indication for PCI was more commonly ST-elevation myocardial infarction in EES patients, balanced by a higher percentage of PCI for stable angina in patients included in the SES arm. Angiographic and procedural characteristics are shown in Table 2. EES patients showed a lower percentage of heavily calcified coronary lesions compared to SES patients. Also, the vascular access site differed significantly, with slightly more radial access in the EES arm. Finally, EES patients were treated more frequently with glycoprotein IIb/IIIa inhibitors compared to patients in the SES arm.

**Table 1:** Baseline characteristics.

	EES (N = 498)	SES (N = 479)	p Value
Age, years	65.3±11.3	65.0±11.2	0.688
Male	353 (70.9)	347 (72.4)	0.589
Risk factors			
Insulin dependent diabetes mellitus	22 (4.6)	29 (6.3)	0.248
Non-insulin dependent diabetes mellitus	28 (5.8)	41 (8.9)	0.072
Hypertension	208 (42.8)	238 (51.0)	0.012
Hypercholesterolemia	249 (53.7)	251 (54.9)	0.701
Family history of cardiovascular disease	263 (55.3)	256 (55.9)	0.843
Current smoker	133 (27.4)	107 (23.2)	0.137
Previous myocardial infarction	109 (22.1)	108 (22.7)	0.829
Previous percutaneous coronary intervention	83 (16.8)	108 (22.5)	0.023
Previous coronary artery bypass grafting	50 (10.1)	72 (15.0)	0.019
Renal insufficiency	53 (11.6)	45 (10.3)	0.542
Indication for percutaneous coronary intervention			
Stable angina	251 (50.4)	275 (57.4)	0.028
Non ST-segment elevation myocardial infarction or unstable angina	179 (35.9)	160 (33.4)	0.404
ST-segment elevation myocardial infarction	68 (13.7)	44 (9.2)	0.028

**Table 2:** Angiographic and procedural characteristics.

	EES (N = 498)	SES (N = 479)	p Value
Target vessel location			0.109
Left main artery	26 (5.3)	15 (3.2)	
Left anterior descending artery	214 (43.4)	184 (38.7)	
Left circumflex artery	106 (21.5)	131 (27.5)	
Right artery	145 (29.4)	144 (30.3)	
Bypass graft	2 (0.4)	2 (0.4)	
Lesion type			0.251
A	24 (4.9)	35 (7.4)	
B1	192 (39.1)	173 (36.5)	
B2	176 (35.8)	158 (33.3)	
C	99 (20.2)	108 (22.8)	
Heavy calcification	65 (13.2)	89 (18.7)	0.019
Chronic total occlusion	18 (3.7)	25 (5.3)	0.229
Bifurcation lesion	116 (23.7)	100 (21.1)	0.338
Visible thrombus	89 (18.1)	72 (15.2)	0.221
Thrombus aspiration	11 (2.3)	7 (1.5)	0.379
Extent of coronary artery disease			0.688
1-vessel	227 (45.7)	208 (43.4)	
2-vessel	171 (34.4)	166 (34.7)	
3-vessel	99 (19.9)	105 (21.9)	
Access site			0.019
Radial	302 (61.1)	255 (53.7)	
Femoral	192 (38.9)	220 (46.3)	
Rotablation	8 (1.6)	4 (0.8)	0.271
Glycoprotein IIb/IIIa blocker	143 (29.2)	106 (22.3)	0.015
Total stent length (mm)	28.1±19.2	28.1±15.8	0.942
Max stent diameter (mm)	3.1±0.6	3.1±0.5	0.199
Multivessel intervention	95 (19.3)	94 (19.7)	0.851
Number of stents/patients	1.50±0.76	1.47±0.76	0.477

**Figure 2:**
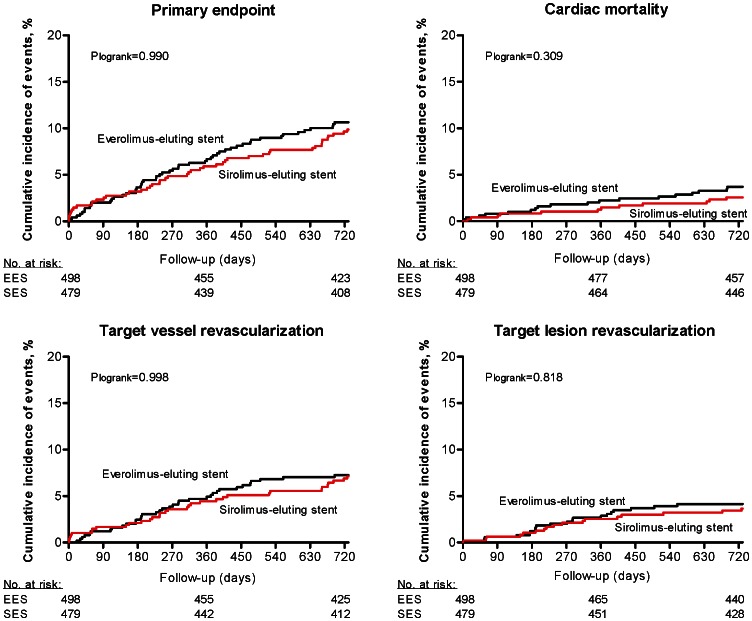
Two-year clinical outcomes. Primary endpoint = composite of cardiac mortality, myocardial infarction and target vessel revascularization.

Clinical outcomes at two years (Table 3, Figure 2) were balanced between patients treated with EES and SES, resulting in a HR of 1.00 (95% CI 0.68–1.48) for the primary endpoint rate. This did not change after adjustment for potential misbalance between the two groups: EES showed a HR of 1.02 (95% CI 0.68–1.53) compared to SES for the primary endpoint rate. Furthermore, per-protocol analysis showed identical results: implantation of EES resulted in a HR of 0.99 (95% 0.81–1.20) for the primary endpoint compared to SES.

**Table 3:** Clinical outcomes at 2 years.

	EES (N = 498)	SES (N = 479)	p Value	Unadjusted HR (95% CI)	Adjusted HR (95% CI)
Major adverse cardiac events*	52 (10.7)	50 (10.6)	0.990	1.00 (0.68–1.48)	1.02 (0.68–1.53)
Mortality					
All-cause	28 (5.7)	22 (4.7)	0.453	1.24 (0.71–2.16)	1.28 (0.72–2.27)
Cardiac	18 (3.7)	12 (2.6)	0.309	1.46 (0.70–3.03)	1.52 (0.72–3.22)
Myocardial infarction	4 (0.9)	8 (1.7)	0.224	0.48 (0.15–1.60)	0.52 (0.16–1.74)
Revascularizations					
Any	57 (11.8)	58 (12.4)	0.768	0.95 (0.66–1.36)	0.92 (0.62–1.35)
Target vessel revascularization	35 (7.3)	34 (7.3)	0.998	0.99 (0.62–1.60)	1.05 (0.65–1.69)
Target lesion revascularization	20 (4.1)	18 (3.9)	0.818	1.08 (0.57–2.04)	1.09 (0.57–2.08)
Stent thrombosis					
Definite	2 (0.4)	4 (0.9)	0.395	0.49 (0.09–2.66)	0.56 (0.10–3.07)
Early (0 to 30 days)	0 (0.0)	1 (0.2)	0.308		
Late (30 days to 12 months)	0 (0.0)	1 (0.2)	0.308		
Very late (>12 months)	2 (0.4)	2 (0.4)	0.971		
Definite/probable	3 (0.6)	6 (1.3)	0.295	0.49 (0.12–1.94)	0.52 (0.13–2.09)

Percentages are cumulative incidences of events based on survival tables. CI = confidence interval; HR = hazard ratio.

* Cardiac mortality, myocardial infarction and target vessel revascularization.

During follow-up, use of dual antiplatelet was similar between the groups. Aspirin (or coumadin when indicated) was used for at least 1 year in 97.8% of EES and 99.3% of SES patients (p = 0.053), while 97.7% of EES and 97.8% of SES patients used aspirin for at least 2 years (p = 0.943). Clopidogrel was used for at least 1 year in 97.3% of EES and 97.3% of SES patients (p = 0.977), while 12.6% of EES and 11.0% of SES patients used clopidogrel at the 2-year follow-up moment (p = 0.445).

## Discussion

In the present open-label, randomized clinical trial, the primary endpoint was comparable between EES and SES during 2-year follow-up. Moreover, secondary endpoints were balanced and definite ST was low in both groups, supporting the safety of DES appliance in clinical practice.

The heightened occurrence of late ST in first generation DES compared to bare-metal stents led to the development of next generation stents in attempt to improve the safety and efficacy of DES [2], [3]. The COMPARE and SPIRIT trials established the superiority of EES over first generation paclitaxel-eluting stents [4], [5]. Major trials comparing EES with SES, the previous golden standard DES, are on-going but so far mostly limited to 1-year follow-up. The current trial compared EES and SES in an all-comer population, which included a high percentage of acute coronary syndromes and complex lesions such as bifurcations and chronic total occlusions. No differences in the primary and secondary endpoints between the 2 limus-based stents were observed during 2-year follow-up.

The findings of this single-center trial are in accordance with the results of the SORT OUT IV (Scandinavian Organization for Randomized Trials With Clinical Outcome IV) Trial [6]. The Danish investigators found no differences in clinical endpoints during 2-year follow-up, with the exception of a lower rate of definite ST in EES patients. ST rates found in SORT OUT IV mirror our results, albeit that the current study was underpowered to detect a difference in ST rates. The largest follow-up available to date exists in the ISAR-TEST-4 (Intracoronary Stenting and Angiographic Results: Test Efficacy of 3-Limus-Eluting Stents-4) trial, showing no differences in outcome up to 3 years [7]. As both arms of the permanent polymer part of the trial consisted of approximately 650 patients, this trial was most likely also underpowered to detect differences in ST. Consistent among all previous trials was the similar efficacy of both stent types. The RESET (Randomized Evaluation of Sirolimus-Eluting Versus Everolimus-Eluting Stent Trial) and the EXCELLENT (Efficacy of Xience/Promus Versus Sirolimus-Eluting Stents in Patients Undergoing Percutaneous Coronary Intervention) trials further support this by showing comparable rates of late luminal loss in these stents during 1-year follow-up [10], [11]. The comparable efficacy is explained by the chemically similar antirestenotic drugs with a virtually identical elution pattern applied in these stents, albeit that the total dose is lower in the EES. The characteristics that set EES apart from SES are the slim 81µm struts covered with biocompatible fluoropolymer. Compared to the 140 µm struts with polyethylene-co vinyl acetate+poly n-butyl methacrylate polymer used in SES, endothelialization is faster after EES implantation. This possibly explains the slight safety benefit of EES over SES as incomplete endothelialization of strut surface is a substrate for stent thrombosis [12]. The important influence of polymer on ST was further emphasized in the LEADERS (Limus Eluted from A Durable versus ERodable Stent coating) trial, which showed an improvement of (very) late ST rates after implantation of stents with a biodegradable polymer compared to conventional SES [13]. The question whether the final solution of long term safety issues will be provided by complete disappearance of the stent structure is currently under investigation in the ABSORB II trial [14].

Additional studies focused on comparison of EES with second generation Resolute zotarolimus-eluting stents. In both the RESOLUTE All Comers trial and the TWENTE trial, no differences in outcome between EES and zotarolimus-eluting stents were observed [15], [16]. Importantly, both stents showed low rates of ST during follow-up, supporting the safety of DES appliance in clinical practice.

### Limitations

There are several limitations that apply to the current study. The randomization sequence was based on date of birth, which may have been the cause of the partial misbalance between the groups, theoretically influencing the outcomes. However, adjustment for baseline differences using cox proportional hazards analyses resulted in only marginal changes in the effect sizes for the individual endpoints, suggesting that the misbalance between the arms was of little influence. Furthermore, the required population size was deduced from the power analysis of the primary study, the XAMI trial. An independent power analysis may have been more accurate. Moreover, the final number of patients was not reached due to inclusion that was slower than anticipated and therefore, the trial was underpowered to discriminate between rare events such as ST. Operators were not blinded to the allocated stent and event adjudication was performed in an unblinded fashion. This reduced the objectivity of the results although the adjudication on consensus basis makes it unlikely that one of the stent types would have been favored. Finally, SES are no longer in clinical use. However, monitoring clinical outcomes of current and previously used stents is necessary to provide further evidence for existing devices and guide future developments of newer generation stents.

## Supporting Information

Checklist S1 **CONSORT checklist.**
(DOC)

Protocol S1 **Trial protocol.**
(PDF)
