# Correction: Two-Year Results of an Open-Label Randomized Comparison of Everolimus-Eluting Stents and Sirolimus-Eluting Stents

**DOI:** 10.1371/annotation/ba8e169c-a2bc-4423-b664-0de7496e453a

**Published:** 2013-09-19

**Authors:** Matthijs A. Velders, Sjoerd H. Hofma, Jan Brouwer, Cornelis Jan de Vries, Michel Queré, Adrianus J. van Boven

The name of the fourth author was spelled incorrectly. The correct name is: Cornelis Jan de Vries. 

