# Supplementary material for: Two-Year Results of an Open-Label Randomized Comparison of Everolimus-Eluting Stents and Sirolimus-Eluting Stents
Source: PLoS One. 2013 Jun 3;8(6):e64424. doi: 10.1371/journal.pone.0064424 (PMC3670908; doi:10.1371/journal.pone.0064424)
Supplement: Protocol S1 — Trial protocol. (PDF) [file pone.0064424.s002.pdf]

Xience V stent vs. Cypher stent

IN ALL MCL PCI PATIENTS

A RANDOMIZED TRIAL

June 2007

Amendment 1

Amendment 1 to Appendixami study version of 24 april 2007.

- Refering to page 7 of protocol extra text:

"The APPENDIXAMI study will stop at the time the XAMI study stops. This includes an early stop reason as indicated by the DSMB".

- Refering to page 18 of protocol STUDY INSURANCE:

"Following the advice of the central Medical Ethical Committee of this study, the RTPO of the MCL hospital, no additional insurance is needed. The patients are insured by the WA hospital insurance at Medirisk".

1. STUDY SUMMARY

Type of Study	Single-centre, prospective, randomized (XienceV versus CYPHER)<br>XAMI APPENDIX
Centres	1 centre
Number of subjects	A number of 2000 patients, for each treatment arm 1000 patients
Purpose of the study	The purpose of this study is to assess the effectiveness of the Xience stent compared to the CYPHER stent in the PCI treatment of **all patients** in the MCL during 2007-2008. This study runs parallel with XAMI study
Primary endpoint	Clinical MACE at 1 year (cardiac mortality, non-fatal myocardial infarction, TVR
Secundary endpoints	• (Sub)-acute stent thrombosis (SAT) at 30 days and late stent thrombosis (LST) at 1, 2 and 3 year.<br>• Clinical MACE at 30 days and 2 and 3 years (cardiac death, non-fatal MI, TVR).<br>• All cause mortality at 1, 2 and 3 year.
Study duration	18 months enrolment, and 36 month follow up

Study design

This is a prospective randomized single-centre registry study designed to assess the clinical performance of the Xience stent in **all MCL PCI patients** compared to CYPHER during 2007 and 2008.

The patients will undergo a clinical examination to assess their clinical status and the occurrence of adverse clinical events before and after the procedure and follow-up inquiry at 30 days, 1, 2 and 3 years after the procedure.

Number of patients

A total number of 2000 patients should be randomized between Xience and CYPHER and enrolled in the study.

Study duration

The duration of the enrolment period will terminate when a total number of 2000 patients will be enrolled.
The estimated study duration is 54 months, i.e. 18 months enrolment and 36 months follow up.

Follow-up methods

Patients will be submitted to clinical examination or assessment at every scheduled follow-up (30 days and 1, 2 and 3 year) by telephone and/or inquiry at referring cardiologist:

	Pre-procedure	Post-procedure	At discharge	1 month follow-up	1,2,3 y follow-up
Physical examination	X	X	X		
Adverse events	X	X	X	X	X
Coronary Angiography	X	X			
Blood Sampling	X	X			

Data Reporting

Data will be recorded on dedicated Case Report Forms.

2. INTRODUCTION AND STUDY RATIONALE

Rationale for use of DES in all comers and ***XAMI appendix***

In the Netherlands DES stents are implanted routinely in 3 centres in a high percentage of patients (Rotterdam x2, Leeuwarden) In the MCL from 2004 the Cypher stent is used routinely. The first clinical results of MCL are encouraging with a low mortality, a low rate of (late) stent thrombosis and re-intervention (rePCI). Results of MCL are competitive to those in the literature. However recent retrospective analyses and registries suggest a higher late risk for stentthrombosis in DES stent patients compared to bare metal stent patients (NEJM 2007). The FDA recently warned against off-label use of DES, which resulted in a drop of DES use by 30%. All this could be due to a shift towards a change in PCI indication (longer lesions, smaller vessels and total occlusions) and the use of DES stents. Randomized prospective data are lacking.
Based on the encouraging results of the MCL the current PCI strategy with DES is however continued, with the exemption that all patients are monitored closely and that follow up is extended. The present single-centre study is an outcome study of all patients treated in the MCL and is part of the XAMI study where there is a focus on AMI patients in a multi-centre setting.
Using randomization the clinical effects of the new generation XienceV stent can be assessed against the golden standard, Cypher stent in an unbiased fashion. Furthermore the data of Xience V stents can be pooled with those of other centres in the Netherlands.

Rationale for use of DES in ***AMI patients***

The benefit of primary Percutaneous Coronary Intervention (PCI) over thrombolytic therapy for acute ST-elevation myocardial infarction (STEMI) has been wel established. Keeley et al. showed in a meta-analysis a clear benefit of PCI, including for patients transported from a non-interventional hospital to an intervention center for primary PCI.[1]
Superiority of stenting versus balloon angioplasty in primary PCI was shown in several randomised trials.[2,3] The benefit in Major Adverse Cardiac Events (MACE: death or reinfarction or Target Vessel Revascularisation (TVR)) in these trials was almost entirely due to reduction in TVR. At 1 year this reduction after stenting was sustained, even compared to patients in whom an optimal "stent-like" result was achieved with balloon angioplasty.[4] No significant reduction in mortality or reinfarction was apparent.[3]

Despite the reduction of TVR in bare metal stents compared to balloon angioplasty, restenosis rates are still significant. In the low risk randomised Stent-PAMI infarct study, 6 months angiographic restenosis rates were 20.3 % for the stented patients and at 1 year ischemic TVR was needed in 16.0 % of patients. In the more contemporary CADILLAC trial, ischemic TVR at 1 year was 13.3%. In these trials only low risk non-shock patients were included.
Suryapranata et al. recently reported the results of 1683 consecutive patients with STEMI (4 % of patients presenting with Killip class 3-4) treated with primary PCI. At 1 year, TVR was performed in 17.3% of patients after stenting and in 19.3 % of patients after balloon

angioplasty.[5] In contrast to the studies reported above, the difference between balloon and stent was not significant in this randomised study. Bare stent thrombosis was seen in 4.8 % of stented patients at 1 year, of which 3.7 % between 0 and 30 days. At 1 year, MACE was 23.1 % in stented patients.
Despite the clear benefit of primary PCI over thrombolysis and the low mortality in several trials, the MACE rate at 1 year is substantial. Most of the MACE is caused by unplanned reinterventions in the infarct related vessel.

The efficacy of drug-eluting stents (DES) to treat coronary artery stenosis in stable patients has been proven in recent trials[6-9].
The use of DES in acute myocardial infarction could be an effective way to decrease the MACE rate after primary PCI. However, the potential risk of higher thrombogenicity has led to cautious use in acute coronary syndromes. Despite early FDA warnings no significant increase in stent thrombosis has been substantiated in stable patients so far, though this is depending on definition and method of data analysis. Premature discontinuation of double anti-platelet therapy is the strongest predictor of stent thrombosis.
The risk of stent thrombosis after DES in STEMI patients has not been studied in large randomised trials, but data from smaller registries and randomized trials has recently been published. Lemos et al. detected no angiographic stent thrombosis at 1 year follow-up in 186 consecutive STEMI patients treated with a sirolimus-eluting stent.[10] Hofma et al. reported the results of 136 consecutive patients treated with Taxus stents for STEMI.[11] 2.9 % subacute stent thrombosis was seen at 30 days and no additional stent thrombosis up to 1 year. In this total DES group (322 patients) incidence of stent thrombosis after 1 year was 1.2 %. This is not higher than rates reported for bare stents in stable patients and compares favourably to the rates reported by Suryapranata et al. for bare stents in STEMI. The STRATEGY trial, a single center primary PCI trial randomising patients to abciximab and bare metal stent or tirofiban and sirolimus-eluting stent showed no subacute stent thrombosis at 30 days in the sirolimus-eluting stent vs. 3 % in the bare stent group (not significant). In both groups no late thrombosis was seen up to 8 months.[12]
The TYPHOON trial randomized 712 patients who underwent primary PCI for STEMI to sirolimus-eluting stent or bare metal stent. Very similar percentages of stent thrombosis of 3.4 % in the sirolimus group vs. 3.6 % in the bare stent group at 1 year were reported, using a liberal definition of stent thrombosis[13]. The PASSION trial, randomizing 619 patients to a paclitaxel-eluting stent or the same uncoated stent in acute myocardial infarction reported similar stent thrombosis incidence in both groups of 1 % at 1 year, using a stricter definition[14].

While stent thrombosis risk does not seem to be higher with DES, a spectacular decrease in TVR was seen in the studies mentioned above. Lemos reported 1.1 % TVR at 1 year in 186 patients treated with sirolimus-eluting stents. Both patients were treated within 48 hours because of persistent complaints with stent edge dissection and incomplete lesion coverage. Thereafter no single patient returned for TVR up to 1 year. Total MACE at 1 year was 9.7 %. The STRATEGY trial reported 7 % TVR in the DES group versus 20 % in the bare stent group at 8 months follow-up. In the TYPHOON trial TVR was 5.6% in the sirolimus patients vs. 13.4 % in the bare stent group at 1 year, with target vessel failure (TVR or recurrent infarction or target-vessel related death) of 7.3 % vs 14.3 % in the control group. For the paclitaxel-coated stent, Hofma reported 6.6 % TVR, mainly caused by a few stent thrombosis patients, who had received complex bifurcation stenting. In this group MACE was 14.7 % at 1 year. In these all-comer groups more than 12 % of patients were in shock

and in 15 % of patients multiple vessels were treated at the index procedure. However, the primary endpoint of cardiac death, recurrent myocardial infarction or TLR at 1 year was not significantly less for the paclitaxel-coated stent compared to the uncoated stent in the PASSION trial (8.8% vs 12.8% p=0.09). It may be argued that the trend is clear but the study was underpowered to show a significant difference.

In conclusion, in early studies using DES for PCI in STEMI, stent thrombosis rates are reassuringly low, at least up to 1 year, and at least comparable with contemporary data with bare stents. It seems advisable to keep procedures simple and, if possible, to avoid bifurcation stenting in the acute setting. A spectacular drop in TVR was reported in the patients treated with DES, thereby reducing the MACE. Bare stents do not decrease mortality and re-infarction rate compared to balloon angioplasty. This is also not to be expected from DES. Despite the fact that larger randomised studies are needed to confirm the data, the use of DES for STEMI seems save and very effective. However, long-term safety of DES for STEMI still has to be confirmed in trials with longer follow-up.

Concerns about delayed healing after DES and thrombosis risk

Despite the favourable clinical data of DES in large trials[6-9] and in patients with acute myocardial infarction[10-14] or acute coronary syndromes[15], delayed healing response[16,17] is a matter of concern. Some increase in late stent thrombosis has been reported during the 2006 ESC and TCT meetings (presentations by E. Camenzind, P. Wenaweser, G. Stone) despite the fact that absolute numbers were depending on definition and analysis method of the raw patient data. Despite the fact that so far the data is not yet very alarming, the very high hazard ratio of stent thrombosis after premature discontinuation of clopidogrel is illustrative for the immature healing response after injury in the first months and maybe up to two years after DES implantation.
Delayed vessel wall healing also implies delayed endothelialization and impaired endothelial function post intervention. Recently, several reports have been published suggesting endothelial dysfunction at least up to 6 months after implantation of sirolimus-eluting stents[18,19].
Possible mechanisms of delayed healing include a hypersensitivity reaction to the non-erodable polymer on the surface of the stent from which local drug delivery is facilitated [20] or a direct effect of the drug on the endothelial cells [18,21].

Possible advantage of Xience V stent over other DES

1.The Xience V stent has very thin stent struts (81 μm) compared to the Cypher stent (134 μm).
It has been shown before for bare metal stents, that strut geometry and thickness influence the vessel wall injury and neointimal proliferation[22,23]. Less extensive vessel wall injury during stenting with thin strut stents may influence the time-table of wound healing response and restoration of endothelial function.
2. This thinner strut thickness and lower crossing profile of the Xience V stent compared to the Cypher stent increases the deliverability success of the stent, especially in tortuous and calcified lesions, as well as in distal lesions. Moreover, lower profile may decrease the risk

of polymer damage during delivery, as was recently shown with scanning microscopy for a Cypher stent of which delivery through another stent had failed[24].

3. The non-erodable two polymers used on the Xience V stent are acrylic and fluoro polymers, biocompatible and already used for years in vascular sutures. They may result in less inflammatory reaction after stent implantation. Less inflammatory response or shorter inflammatory reponse may also have a beneficial influence on vascular healing and neointimal proliferation.

4. In animal models, delayed arterial healing and poor re-endothelialization was demonstrated after oral everolimus in a rabbit model of iliac artery stent implantation. Despite the fact that low dose everolimus did not show these effects, the suppression of neointima was similar in the high and low dose group[25]. This suggests a wide therapeutic window and the possibility to keep the detrimental effects on vascular wall healing limited together with effective suppression of neointimal proliferation. In fact, recently, Finn et al. demonstrated almost complete endothelialization of a Xience V stent with even tight intercellular junctions, 14 days after implantation in a porcine coronary artery[26]. This was in contrast to poor endothelialization of the TAXUS stent in the same model.

The issues raised above provide the rationale for testing the performance of the Xience V stent in patients with acute myocardial infarction and in all comers.

In this protocol of the **XAMI APPENDIX** study the Xience V stent will be tested against the gold standard of drug-eluting stents at this moment, the Cypher (sirolimus-eluting) stent. This study runs parallel with XAMI study.

The APPENDIXAMI study will stop at the time the XAMI study stops. This includes an early stop reason as indicated by the DSMB.

References:

1. Keeley EC, Boura JA, Grines CL. Primary angioplasty versus intravenous thrombolytic therapy for acute myocardial infarction: a quantitative review of 23 randomised trials. *Lancet* 2003;**361**:13-20.
2. Stone GW, Grines CL, Cox DA, et al., for the Controlled Abciximab and Device Investigation to Lower Late Angioplasty Complications (CADILLAC) investigators. Comparison of angioplasty with stenting, with or without abciximab, in acute myocardial infarction. *N Engl J Med* 2002;**346**:957-66.
3. Zhu MM, Feit A, Chadow H, et al. Primary stent implantation compared with primary balloon angioplasty for acute myocardial infarction: a meta-analysis of randomized clinical trials. *Am J Cardiol* 2001;**88**:297-301.
4. Cox DA, Stone GW, Grines CL, et al. Outcome of optimal or "stent-like" balloon angioplasty in acute myocardial infarction: The CADILLAC trial. *J Am Coll Cardiol* 2003;**42**:971-7.
5. Suryapranata H, De Luca G, van't Hof AWJ, et al. Is routine stenting for acute myocardial infarction superior to balloon angioplasty? A randomised comparison in a large cohort of unselected patients. *Heart* 2005;**91**:641-45.
6. Morice MC, Serruys PW, Sousa JE, et al., for the RAVEL study group. A randomized comparison of a sirolimus-eluting stent with a standard stent for revascularization. *N Engl J Med* 2002;**346**:1773-80.

7. Moses JW, Leon MB, Popma JJ, et al., for the SIRIUS investigators. Sirolimus-eluting stents versus standard stents in patients with stenosis in a native coronary artery. *N Engl J Med* 2003;**349**:1315-23.
8. Colombo A, Drzewiecki J, Banning A, et al., for the TAXUS II study group. Randomized study to assess the effectiveness of slow- and moderate-release polymer-based paclitaxel-eluting stents for coronary artery lesions. *Circulation* 2003;**108**:788-94.
9. Stone GW, Ellis SG, Cox DA, et al., for the TAXUS-IV investigators. A polymer-based, paclitaxel-eluting stent in patients with coronary artery disease. *N Engl J Med* 2004;**350**:221-31.
10. Lemos PA, Saia, F, Hofma SH, et al. Short- and long-term clinical benefit of Sirolimus-eluting stents compared to conventional bare stents for patients with acute myocardial infarction. *J Am Coll Cardiol* 2004;**43**:704-8.
11. Hofma SH, Ong ATL, Aoki J, et al. One year clinical follow-up of paclitaxel eluting stents for acute myocardial infarction compared with sirolimus eluting stents. *Heart* 2005;**91**:1176-80.
12. Valgimigli M, Percoco G, Malagutti P, et al. Tirofiban and sirolimus-eluting stent vs abciximab and bare-metal stent for acute myocardial infarction. A randomized trial. *JAMA* 2005;**293**:2109-2117.
13. Spaulding C, Henry P, Teiger E, et al. Sirolimus-eluting versus uncoated stents in acute myocardial infarction. *N Engl J Med* 2006;**355**:1093-104.
14. Laarman GJ, Suttorp MJ, Dirksen MT, et al. Paclitaxel-eluting versus uncoated stents in primary percutaneous coronary intervention. *N Engl J Med* 2006;**355**:1105-13.
15. Moses JW, Mehran R, Nikolsky E, et al. Outcomes with the paclitaxel-eluting stent in patients with acute coronary syndromes: analysis from the TAXUS-IV trial. *J Am Coll Cardiol.* 2005;**45**:1165-71.
16. Suzuki T, Kopia G, Hayashi S, et al. Stent-based delivery of sirolimus reduces neointimal formation in a porcine coronary model. *Circulation* 2001;**104**:1188-1193.
17. Drachman DE, Edelman ER, Seifert P, et al. Neointimal thickening after stent delivery of paclitaxel: change in composition and arrest of growth over six months. *J Am Coll Cardiol.* 2000;**36**:2325-32.
18. Hofma SH, van der Giessen WJ, van Dalen BM, et al. Indication of long-term endothelial dysfunction after sirolimus-eluting stent implantation. *Eur Heart J* 2006;**27**:166-170.
19. Togni M, Windecker S, Cocchia R, et al. Sirolimus-eluting stents associated with paradoxic coronary vasoconstriction. *J Am Coll Cardiol* 2005;**46**:231-236.
20. Nebeker JR, Virmani R, Bennett CL et al. Hypersensitivity cases associated with drug-eluting coronary stents. *J Am Coll Cardiol* 2006;**47**:175-81.
21. Lerman A, Eeckhout E. Coronary endothelial dysfunction following sirolimus-eluting stent placement: should we worry about it? Editorial. *Eur Heart J* 2006;**27**:125-126.
22. Hofma S.H, Whelan DMC, van Beusekom HMM, Verdouw PD, van der Giessen WJ. Increasing arterial wall injury after long-term implantation of two types of stent in a porcine coronary model. *Eur Heart J* 1998;**19**:601-609.
23. Kastrati A, Mehilli J, Dirschinger J, et al. Intracoronary stenting and angiographic results: strut thickness effect on restenosis outcome(ISAR-STEREO) trial. *Circulation* 2001;**23**:2816-21.
24. Kuriyama N, Kobayashi Y, Nakayama T, Kuroda N, Komuro I. Damage to polymer of a sirolimus-eluting stent. *Circulation* 2006;**114**:e586-587.

25. Farb A, John M, Acampado E, et al. Oral everolimus inhibits in-stent neointimal growth. *Circulation* 2002;**106**:2379-84.
26. Finn AV, Nakazawa G, Joner M, Virmani R. Everolimus eluting stents: beyond targeting restenosis! *Eurointerv* 2006;**2**:277-279.

3. INSTITUTION

CENTRE INVOLVED IN THE STUDY:

- **Medisch Centrum Leeuwarden**

Henri Dunantweg 2, 8934 AD Leeuwarden

The Netherlands

Tel: +31 58 2866666

Fax: +31 58 2867840 (planning secretary cathlab)

Principal Investigator: Dr. A.J. Van Boven

Investigators: Dr S.H. Hofma, Dr. J.Brouwer, Drs. M Queré, Drs. C.J. de Vries

4. DESCRIPTION OF DEVICES

4.1 XienceV stent

The Xience V stent system (Abbott Vascular, Santa Clara, CA, USA) is composed of two components: a) the Xience Everolimus Eluting stent (an L605 Cobalt Chromium (CoCr) alloy stent) premounted on a delivery system and b) a drug eluting coating (a formulation of two polymers and the anti-proliferative drug everolimus, a sirolimus-analoque.

The stent is very flexible with thin struts but good radial strength thanks to the CoCr material. The strut thickness is 81 µm (0.0032 inch).

For use of the device please consult the instructions for use.

4.2 *CYPHER DES*

The Cypher stent (Cordis Co, Warren, NJ, USA) is a 316 L stainless steel metal stent with a polymer coating eluting the anti-proliferative drug sirolimus. The strut thickness of the BX-Velocity stent, the platform of the Cypher stent is 134 μm (0.0055 inch).
For use of the device please consult the instructions for use.

5.STUDY DESIGN

This is a prospective randomized study designed to compare the effectiveness and safety, defined as clinical MACE at 12 months follow up, of the Xience Stent with the CYPHER stent in all patients treated for PCI in MCL. In order to study the long-term safety of the both DES, follow-up is extended to 3 years.

5.1.1 Endpoint of the Study

The **primary endpoint** of the study is to assess the 12 month MACE (cardiac death, non-fatal MI, TVR) in all MCL PCI patients comparing the Xience stent to the CYPHER stent.
The **secondary endpoints** are:

- (Sub)-acute stent thrombosis (SAT) at 30 days and late stent thrombosis (LST) at 1, 2 and 3 year. (Definite and Probable, according to ARC definition). Possible stent thrombosis will be reported separately.
- Clinical MACE at 30 days and 2 and 3 years (cardiac death, non-fatal MI, TVR).
- All cause mortality at 1,2 and 3 year.
- Inability to deliver Cypher or Xience stent (cross over)

5.1.2 Definitions

Stent thrombosis:
According to the recently proposed ARC (Academic Research Consortium) definitions (Cutlip et al. TCT 2006).

- **Definite:** When confirmed by angiography (angiographic documentation of target vessel occlusion or visual thrombus) or when pathologic confirmation of acute thrombosis in ACS patient is made.
- **Probable:** any unexplained death within 30 days or target vessel MI without angiographic confirmation of thrombosis or other identified culprit lesion.
- **Possible:** any unexplained death after 30 days.

- **Timing:** Early: Acute < 24 hours
Subacute 1-30 days
Late: 30 days – 1 year
Very Late: > 1 year

Myocardial infarction:

- Re-infarction is defined by recurrent symptoms and/or new electrocardiographic changes, in association with re-elevation of the creatine kinase (CK) of > 1.5 times the previous value with elevation of CK-MB, if within 48 h, or > 3 times the upper normal limit, if after 48 h from the index infarction.
- More than 5 times the upper limit of normal CK is required for the diagnosis of myocardial infarction after bypass surgery.

Target vessel revascularization(TVR):

- TVR is defined as any repeat percutaneous intervention or by-pass grafting of the target vessel previously treated successfully with the stent.

Target lesion revascularization (TLR):

- TLR is defined as any repeat percutaneous intervention or by-pass grafting of the target lesion previously treated successfully with the stent or the 5 mm proximal or distal to this initial lesion.

Major Adverse Cardiac Events (MACE):

- MACE is defined as any event during follow-up in hierarchical order: cardiac death, non-fatal re-infarction or TVR.

5.2 *Patients selection criteria*

The patient population must be geographically stable and able to perform the follow-up examinations at or by the Investigation Centre. Patients who are not willing or unable to participate in the follow-up will not be enrolled in the study.

5.2.1 Number of patients

A total number of 2000 consecutive patients fulfilling the inclusion/exclusion criteria and willing to sign the informed consent can be enrolled in the trial.

This study is a clinical outcome study in 2000 all comers during the period 2007-2008 and runs parallel with the XAMI trial in which 800 patients are included. Since all types of outcome are expected no sample size calculation is performed.

5.2.2 Study duration

The duration of the enrolment period will terminate when a total of 2000 patients will be enrolled and is estimated to last 18 months.

With a follow-up period of 36 months, the total study duration will be 54 months.

5.2.3 Enrolment criteria

Inclusion criteria

1) Patient is willing to sign the informed consent
2) Patient is willing to perform all follow-up examinations as required by the protocol
3) Patient eligible for coronary revascularization intervention by PCI and stenting
4) Patient is located in a geographic area that will enable contact by the study site for follow-up.

Exclusion criteria

1) Patients who are minor, intravenous drug abusers, alcohol abuser, prisoners or unable to give informed consent

2) Patients who are allergic to everolimus or sirolimus
3) Patients with known intolerance or contra-indications to treatment with acetylsalicylic acid or clopidogrel
4) Patients in whom the cardiologist judges that stent implantation is not possible, realistic or justified.

6. CONDUCT OF THE CLINICAL STUDY

6.1 GENERAL ASPECTS.

The study will be performed in accordance with the European Regulation EN 14155 part 1 and 2. Respect of patient's rights will be granted during the trial phase according to the most recent version of the Helsinki Declaration.

6.2 PREPROCEDURAL SCREENING.

The patients are screened for inclusion and exclusion criteria before offering enrolment in the study. The patient should be informed on the study objectives and the required post procedure follow-up. The patient will provide witnessed oral consent before and sign the informed consent before or after the procedure.

Pre-procedure following is collected:

1. Demographic documentation
2. Clinical examination and clinical history
3. Blood analysis

The patient is considered enrolled in the trial when all selection criteria are met and the randomization envelop has been opened **(after the diagnostic angiogram).**

7. STUDY PROCEDURES AND METHODS

Direct stenting or pre-dilatation is at the discretion of the operator.

7.1 Percutaneous access

Preparation of the access site and percutaneous access should be performed according to the normal hospital practices. Heparin should be administered intravenously to obtain an ACT of 300 sec or more.

7.2 Diagnostic angiographic examination

The diagnostic angiographic examination should be performed in order to identify the vessels affected by atherosclerotic lesions.

7.3 Randomization

Randomization will be done by closed envelopes. The investigator chooses the envelope to the following sequential number available. The randomization code should be recorded on the CRF and notification form.

7.4 Stent implant

The stent should be selected on the basis of the lesion length. The stent length must exceed the target lesion length by 5 mm (2,5 mm on each site). Direct stenting or pre-dilatation is at the discretion of the operator.

If multiple stents need to be implanted, the stent belongs to the same group as previously randomized.

Residual stenosis, after the stent implant, must be less then 30%. In those cases where the result is not optimal it is requested to postdilate the stent.

The implant procedure is considered successful if the residual stenosis is less than 30% with TIMI flow III.

If there are coronary dissections at the end of the stenting procedure, they must be treated with an additional stent from the same randomization group.

When stent implantation is not successful, cross-over to the other stent of this study or bare stent or balloon only, is allowed.

8. MEDICATION

The medication guidelines, described in the protocol, should be preferably followed unless a different therapeutic treatment is necessary.

Pretreatment with ASA and clopidogrel is according to local protocol.

8 tablets of 75 mg clopidogrel should be given before angiography or as soon as possible in those patients who did not receive clopidogrel the day before.

Unfractionated heparin should be used at the beginning of the procedure. Target ACT 300 sec without abciximab or > 200 sec with concommitant use of abciximab.

ASA 80 mg or Ascal 100 mg (lifetime) together with clopidogrel 75 mg a day **(for 1 year !!)** is strongly advised. In case of clear indication for oral anticoagulants (OAC), this should be

combined with clopidogrel for 1 year. Also adding ASA for the first 2 weeks is advised. Less clear indication for OAC should be reconsidered and if possible and acceptable OAC should be stopped and changed into ASA and clopidogrel after the procedure.

9. FOLLOW-UP

After discharge, patient follow-up will be performed after 30 days and 1,2,3 year by questionnaires. Repeat angiography will only be performed clinically driven by symptoms or signs of ischemia or when deemed necessary by the cardiologist. Information about in-hospital outcomes will be obtained from our institutional electronic clinical database and by review of the hospital records for those discharged to referring hospitals. Post-discharge survival status will be obtained from the Municipal Civil Registries at 1 month and 1, 2, 3 year. All repeat interventions (surgical and percutaneous) and re-hospitalizations will prospectively be collected during follow-up. Questionnaires regarding anginal status, intercurrent events and medication use will be discussed with all living patients by telephone at 12 months. Referring physicians and institutions will be contacted for additional information if required.

10. ORGANIZATION AND MONITORING

The clinical investigation plan requires that the investigator performs the clinical investigation on the patients as also the assessment of the patient's clinical status. The clinical follow-up will be performed before hospital discharge of the patient.

Patient data will be collected on Case Report Forms (CRFs). Data will be collected for the required study contacts according to the CRF Schedule. The Investigator will be required to sign each form to indicate the authenticity of the data submitted.

11. RISKS ASSOCIATED AND ADVERSE CLINICAL EVENTS.

The potential risks related to this study are those typically reported for other coronary stents. These risks include, but are not limited to: possible occlusion of the stented vessel, death, myocardial infarction and need for target lesion revascularization, coronary artery perforation or dissection, peripheral bleeding, artery trauma at the

access site, stent embolization, cardiac arrhythmias due to drug side effects, hypersensitivity to the drugs or polymers on the stent.

ADVERSE CLINICAL EVENTS

Adverse clinical event means any unpredicted clinical complication, whether or not it is device related.

Unpredicted adverse event: any clinical event occurring to the patient, related to the device, not reported in the instructions for use.

Severe adverse events: any clinical event occurring to the patient, that is fatal, life-threatening, debilitating or results in prolongation of the hospitalization period. This includes but is not limited to: death, myocardial infarction, revascularization of the target vessel, severe bleeding.

All adverse events, unpredicted and severe, related to the device under evaluation must be reported by fax to the study monitor within 24 hours of their occurrence.

12. ETHICS

To protect the rights and welfare of the patients, this clinical study will be conducted in conformance with the Declaration of Helsinki, the laws and regulations of the country, and hospital requirements, whichever affords greater protection to the patient. Patients will be fully informed of benefits/risks of their participation to the study, the benefits and risks of the clinical procedure to be followed and the follow-up examinations described in the study protocol, according to ISO 14155.

13. ETHICAL COMMITTEE APPROVAL

The Principal Investigator must submit the study protocol to the local ethical committee to have the approval to proceed to the patients' enrolment and randomisation. The Ethical Committee, after verifying that the study protocol meets patients' needs and rights according to the Declaration of Helsinki will notify to the Investigator a formal approval to conduct the study.

Site principal investigator is responsible to file a copy of the Ethical Committee Approval.

14. INFORMED CONSENT FORM

Patients planned to be enrolled in the study must be carefully informed about their participation into a randomised study. A patient can be considered enrolled in the study once his clinical conditions meet the enrolment criteria and if he/she signs the informed consent form (before the procedure or when not feasible after the procedure with oral consent before procedure). This study is not being used for regulatory approval, but a therapeutic protocol using already approved devices. The Investigator is responsible to collect and file the signed informed consent forms.

Patients unable to be followed on-site should not be included in the study.

15. CONFIDENTIALITY

Individual patient medical information obtained as a result of this study will be considered confidential and disclosure to third parties is prohibited. With appropriate patient authorization, medical information may be given to the patient's personal physician or to other medical personnel responsible for his/her welfare.

The data handling ensures compliance to applicable Personal Data Protection Laws and regulations.

16. STUDY INSURANCE

Following the advise of the central Medical Ethical Committee of this study, the RTPO of the MCL hospital, no additional insurance is needed. The patients are insured by the WA hospital insurance at Medirisk.

17. RESPONSIBILITY AND INITIAL OBJECTIVES

The Investigator and co-Investigators must collect the data on the CRF and file the informed consent.

The disclosure of results, including conference presentations and publications in journals, will follow the study agreement between the parties.
